# Glucagon-like peptide-1: effect on pro-atrial natriuretic peptide in healthy males

**DOI:** 10.1530/EC-13-0087

**Published:** 2014-01-23

**Authors:** Jeppe Skov, Jens Juul Holst, Jens Peter Gøtze, Jørgen Frøkiær, Jens Sandahl Christiansen

**Affiliations:** 1 Department of Endocrinology and Internal Medicine Aarhus University Hospital Norrebrogade 44DK-8000, Aarhus Denmark; 2 Novo Nordisk A/S DK-2880, Bagsvaerd Denmark; 3 NNF center for Basic Metabolic Research, Department of Biomedical Sciences The Panum Institute, University of Copenhagen DK-2200, Copenhagen Denmark; 4 Department of Clinical Biochemistry, Rigshospitalet University of Copenhagen Blegdamsvej 9DK-2100, Copenhagen Denmark; 5 Department of Clinical Physiology and Molecular Imaging Aarhus University Hospital Aarhus Denmark; 6 Department of Clinical Medicine Aarhus University DK-8000, Aarhus Denmark

**Keywords:** glucagon-like peptide-1, atrial natriuretic peptide, natriuresis, proANP, proBNP, kidney, heart rate, blood pressure

## Abstract

The antihypertensive actions of glucagon-like peptide-1 (GLP1) receptor agonists have been linked to the release of atrial natriuretic peptide (ANP) in mice. Whether a GLP1–ANP axis exists in humans is unknown. In this study, we examined 12 healthy young males in a randomized, controlled, double-blinded, single-day, cross-over study to evaluate the effects of a 2-h native GLP1 infusion. Plasma proANP concentrations were measured by an automated mid-region-directed proANP immunoassay and N-terminal pro B-type natriuretic peptide (BNP) on Roche Modular E170. Urine was collected for measurements of sodium excretion. Although GLP1 infusion increased the urinary sodium excretion markedly, there were no significant changes in either proANP or proBNP concentrations. When GLP1 infusion was stopped, sodium excretion declined rapidly. As proANP concentration reflects ANP secretion, our data could not confirm the existence of a GLP1–ANP axis in humans. Especially, the natriuretic effects of GLP1 seem unlikely to be mediated exclusively via ANP.

## Introduction

Glucagon-like peptide-1 (GLP1) is a gut-derived incretin hormone with multiple actions in addition to control of glucose homeostasis (1). Synthetic GLP1 receptor (GLP1R) agonists lower blood pressure in patients with type 2 diabetes through mechanisms not fully comprehended (2). The antihypertensive effects seem non-acute in nature (3) but sets in before substantial weight loss (4). GLP1 has natriuretic effects in humans (5, 6) and some studies suggest that GLP1 has vasorelaxant effects (7).

A recently published article by Kim *et al*. (8) reports that atrial natriuretic peptide (ANP) mediates the antihypertensive effects of GLP1 in mice. The authors convincingly demonstrate that GLP1R activation stimulates ANP release from atrial cardiomyocytes and that GLP1 exclusively acts via ANP to induce vasodilatation and natriuresis (8). The findings, therefore, define a novel GLP1–ANP axis and expand our view on GLP1-induced cardiovascular and renal actions.

To explore whether this GLP1–ANP axis exists in humans, we measured plasma proANP concentrations in healthy males exposed to a natriuresis inducing native GLP1 infusion.

## Subjects and methods

### Design

The study was performed using a randomized, double-blinded, placebo-controlled, single-day cross-over design.

### Subjects

Twelve healthy male subjects were studied. Mean age was 23.4±3.1 years (s.d.) and mean BMI was 23.0±2.0 kg/m^2^ (s.d.). Each volunteer provided written informed consent. The protocol was approved by the local Human Ethics Research Committee and carried out in accordance with the Helsinki Declaration.

All volunteers had a normal physical examination as well as normal blood and urine screenings. None were on medication or had any history of critical medical conditions. Fourteen subjects were screened, one withdrew after screening and one was excluded after the study because of fluid retention due to impaired urinations.

### Protocol

The primary study objective was to investigate GLP1-induced effects on kidney function and the procedures and results regarding this part have been previously described (6). In summary, the protocol involved blood sampling, infusions of radioactive isotopes, and a prior ingestion of small doses lithium.

Volunteers arrived at the clinic at 0715 h after an overnight fast. They were placed in bed where they stayed supine during the study day. Catheters were placed in each antecubital vein, one for blood sampling, the other for infusion.

At ∼0730 h, an isotonic 0.9% saline infusion was started at a rate of 750 ml/h and continued throughout the study to stimulate urine production. At 0900 h a 2-h infusion of either GLP1, dissolved in 0.9% saline containing 1% human albumin, or the solvent alone (placebo) was initiated. This was followed by a 2-h washout period before infusion of the placebo and GLP1 solutions for the next 2 h. The GLP1 infusion rate was 1.25 pmol/kg per min.

The two solutions were prepared by the physician in charge and afterwards blinded to both physician and volunteers allowing for the double-blinded study design. Blood samples were drawn every 20 min, resulting in a total collection of ∼300 ml blood during the entire study day. Urine was collected every 20 min by voiding in a bottle. Blood pressure and heart rate (HR) were measured every 20 min throughout the day.

### Materials

Synthetic human GLP1(7–36) amide was obtained from Bachem (Weil am Rhein, Germany).

### Assays

GLP1 plasma concentrations were measured by a RIA method as described earlier (6). For measurement of plasma proANP, we used an automated proANP immunoassay (BRAHMS, Hennigsdorf, Germany) with targeted epitopes in the mid-region (MR) of the precursor (9). Unlike ANP, MR-proANP is well preserved in plasma (10). The intra-assay coefficient of variation (CV) from the manufacturer is <2.5% in the relevant molar range (20–1000 pmol/l); the limit of detection is 2.1 pmol/l, and the limit of quantitation is listed as being 4.5 pmol/l. In our laboratory, the working interassay CV in the current molar range was <2% (*n*=60). The MR-proANP assay has also been compared with an in-house gold standard proANP assay, with excellent correlation in the low range (11).

N-terminal pro B-type natriuretic peptide (BNP) was measured on Roche Modular E170 using a previously well-described method (12). The limit of detection was 0.6 pmol/l and values below this limit were set to 0.5 pmol/l. Urinary sodium concentration was measured by the accredited laboratory of Aarhus University Hospital.

### Statistical analysis

A mixed model of repeated measures ANOVA was applied (subject and subject×period as random variables; group, time, and group×time as fixed variables). The *P* value of the interaction group×time is reported throughout. The proBNP data were logarithmically transformed before analyses. A *P*<0.05 is considered statistically significant. STATA, version 11.1 (StataCorp., College Station, TX, USA) was used for calculations.

## Results

**Figure 1:**
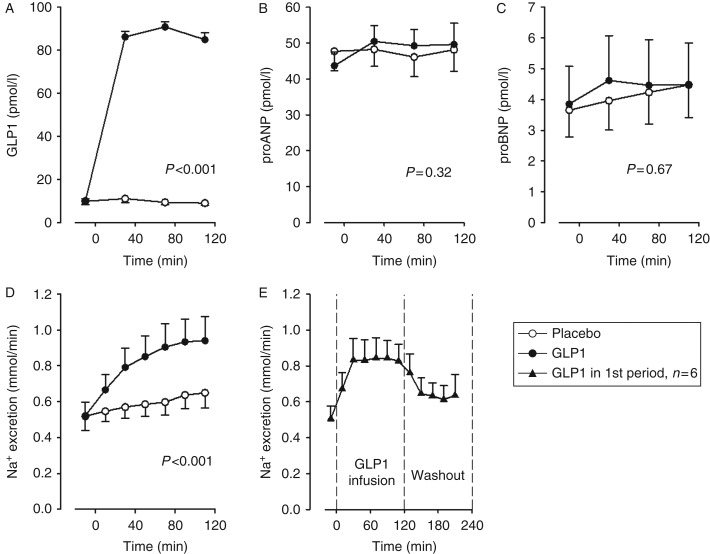
GLP1 infusion increased plasma GLP1 concentration nearly tenfold, (A) but this had no significant effect on plasma proANP (B) or proBNP (C) concentrations despite a marked increase in urinary sodium excretion (D). Sodium excretion rapidly declined when GLP1 infusion stopped (E). Open circles represent values from the placebo period; closed circles from the GLP1 period; and triangles represent data from subjects randomized to GLP1 in the first intervention period (*n*=6). Statistical significance was determined by ANOVA. Data are shown as mean and error bars represent s.e.m.

When infusion of native GLP1 1.25 pmol/kg per min started, the baseline GLP1 concentration increased nearly tenfold and remained elevated until the infusion was terminated (*P*<0.001). The urinary sodium excretion increased ∼40% (*P*<0.001) during GLP1 infusion but rapidly returned toward baseline when infusion stopped (as shown for the group randomized to GLP1 in the first intervention period, *n*=6). GLP1 infusion did not affect proANP plasma concentration (*P*=0.32; see Fig. 1). Both proANP and proBNP concentrations increased slightly over time independent of the order of GLP1 and placebo infusions.

**Figure 2:**
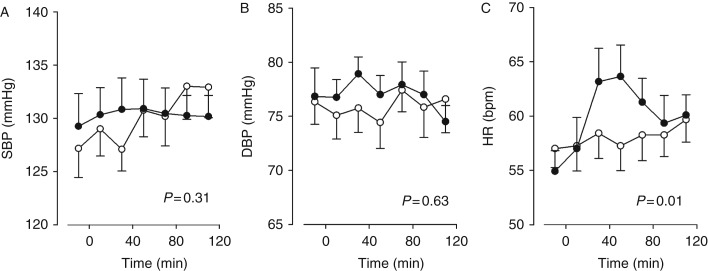
GLP1 infusion had no acute effect on either SBP (A) or DBP (B) but there was a transient increase in heart rate (C). Open circles represent values from the placebo period and closed circles from the GLP1 period. Statistical significance was determined by ANOVA. Data are shown as mean and error bars represent s.e.m.

There was no acute impact on either systolic blood pressure (SBP; *P*=0.31) or diastolic blood pressure (DBP; *P*=0.63), but HR transiently increased by approximately six beats per minute (*P*=0.01). The effect on vital parameters has previously been published (6) (see Fig. 2).

## Discussion

We have shown that an almost tenfold increase in GLP1 concentration, which markedly induced natriuresis, was incapable of increasing plasma proANP or proBNP concentrations in healthy males. As proANP concentration reflects ANP secretion, the result questions the existence of a functional GLP1–ANP axis in humans. Especially the natriuretic effect seems unlikely to be mediated exclusively via ANP.

### GLP1 does not increase proANP concentration

Despite a robust and sustained increase in GLP1 concentrations during the 2-h GLP1 intervention, we were unable to detect an increase in proANP plasma concentration.

Plasma proANP is today used clinically as a surrogate marker for cardiac ANP secretion. The prohormone has a longer half-life in the circulation, shows smaller fluctuations, and the peptide is better preserved in plasma samples (10).

The half-life of ANP is <5 min, and while the exact half-life of proANP is unknown, we have seen 50% reduction of the proANP concentration *in vivo* within 60 min (13). This should facilitate our ability to detect a rise in the secretion of ANP (with its synchronous and equimolar secretion of proANP).

In the article by Kim *et al*. (8), the majority of their numerous experiments were performed with the GLP1R agonist liraglutide. However, by using GLP1R knockout mice, they showed that ANP release indeed depended on GLP1R activations; in addition, exendin-4, native GLP1, as well as a meal (endogenous GLP1) were capable of increasing ANP concentrations.

We would therefore expect native GLP1 to stimulate ANP secretion in humans if the same mechanisms were present. As GLP1 failed to increase proANP levels, we conclude that either the GLP1–ANP axis is solely a mouse phenomenon or that a GLP1 induced release of ANP in humans must be minor or very transient.

A recent paper exploring the effect of a meal on plasma proANP concentrations in humans (13) also speaks against a major effect of GLP1 on ANP secretion. Six overnight fasted healthy young individuals were given a standardized meal and proANP was measured repeatedly for the first three postprandial hours. The meal was found to significantly reduce proANP concentrations. Since a meal is the physiological stimulus for GLP1 secretion we would expect the meal to increase proANP if a GLP1–ANP axis existed in humans.

### No effect of GLP1 on proBNP

We also measured N-terminal-proBNP as a marker of BNP secretion, but found no effect in response to GLP1 infusion. Kim *et al*. (8) did not either find BNP to be a mediator of GLP1-actions in mice. It seems therefore reasonable to conclude that BNP is not part of an important gut–heart axis.

### ANP is not exclusively mediating GLP1's natriuretic actions

We found infusion of native GLP1 to increase the urinary sodium excretion markedly in humans (Fig. 1D). Further, the increase in natriuresis was sustained during the entire period of GLP1 infusion, but rapidly returned toward baseline when infusion was stopped (Fig. 1E). As there were no changes in proANP concentrations, the finding is inconsistent with ANP being an essential mediator of GLP1's natriuretic actions in humans.

Kim *et al*. (8) found liraglutide to markedly increase natriuresis in mice and thereby confirmed an earlier finding of liraglutide-induced diuresis in rats (14). Liraglutide had no effect on natriuresis in ANP knockout mice, indicating that ANP is required for the natriuretic actions of GLP1R agonists (8). The different GLP1R agonists often induced an initial peak in ANP level, which disappeared within the first hour (8). This was especially the case during the light-on periods. We found the increased urinary sodium excretion to be sustained under the GLP1 infusion and hereafter to decrease rapidly. Therefore, it is unlikely that the effect was caused by a hypothetical initial ANP peak not reflected by increases in proANP concentrations.

There may, however, be important differences between native GLP1 and liraglutide with respect to renal actions. The natriuretic effect has been hypothesized to depend on activation of GLP1Rs located in the proximal tubules (15). While GLP1 is freely filtered and partly cleared in the kidneys, only minimal amounts of liraglutide pass the glomerular filtration barrier (16). If GLP1R activation depends on GLP1R agonists located in the tubular lumen, liraglutide may not be functioning. Though liraglutide induces natriuresis in rodents (8, 14), to our knowledge, it has never been reported whether liraglutide actually has natriuretic properties in humans.

ANP infusion markedly increases glomerular filtration rate in rodents (17, 18) and humans (19, 20). GLP1 infusion also strikingly increases glomerular filtration rate in rodents (15, 21) but interestingly not in humans (5, 6). This difference could be explained by a GLP1–ANP axis which is only functional in rodents.

### No acute effect of GLP1 on blood pressure

We did not find native GLP1 infusion to acutely affect blood pressure in healthy males (Fig. 2), which we have also prior reported (6). Kim *et al*. (8) found different GLP1R agonists to reduce blood pressure but only in mice which were hypertensive secondary to an angiotensin II infusion or to transaortic constriction, and the effects were most pronounced during the light-off periods. Our findings, therefore, are not in contrast, because the volunteers were normotensive. Further, the results are also in line with other human studies showing no acute effect of GLP1R agonists on blood pressure (3). A GLP1-induced increase in HR (Fig. 2C) has been reported in both rodents and humans, although usually not reported to be transient. Notably, Kim *et al*. (8) found that the positive chronotropic effect of GLP1R agonists was independent of ANP.

### Implications for proANP as a biomarker of heart failure

Mid-regional proANP has been introduced as a heart failure biomarker (22). Kim *et al*. (8) found GLP1R agonists to markedly increase ANP levels in mice but found no effect on BNP. This indirectly raises the question whether proANP is a valid biomarker of heart failure in patients treated with GLP1-based products or if derives of BNP are better biomarkers in these cases. Diabetes *per se* is known to increase the risk of heart failure, and rising numbers of patients are now treated with GLP1 products which make the concern for false positive results highly relevant.

Our data showed no impact on proANP or proBNP after GLP1 infusion. Consequently, we have no reason to believe that either proANP or proBNP are inadequate biomarkers of heart failure in patients treated with GLP1-based products. The question, however, demands further investigations in type 2 diabetes patients treated with GLP1R agonists for longer periods.

The major limitation of this study is that it was not originally designed to evaluate a GLP1–ANP axis and the proANP analyses were performed *post-hoc*. Further, we did not measure ANP itself because our blood samples were not properly pretreated to allow for acceptable analyses of this highly unstable peptide. High saline infusion rates and frequent blood sampling may have induced period effects.

In conclusion, our data cannot confirm the existence of a GLP1–ANP axis in humans and we find especially GLP1's natriuretic effect unlikely to be caused exclusively via increased ANP secretion. The topic is important because increasing numbers of patients are treated with GLP1-based products which make cardiovascular side effects an obvious topic of interest. Dedicated studies are needed to further explore the GLP1–ANP relationship in humans.
